# Determinants of Maternal Death in a Pastoralist Area of Borena Zone, Oromia Region, Ethiopia: Unmatched Case-Control Study

**DOI:** 10.1155/2019/5698436

**Published:** 2019-01-20

**Authors:** Jarso Sara, Yusuf Haji, Achamyelesh Gebretsadik

**Affiliations:** ^1^Borena Zone Health Office, Oromia Regional State, Borena, Ethiopia; ^2^Borena Zone Health Department, Hawassa University, Hawassa Ethiopia, School of Public Health, Hawassa, Ethiopia

## Abstract

**Background:**

Globally, more than 830 maternal deaths happen daily, and nearly, all of these occur in developing countries. Similarly, in Ethiopia, maternal mortality is still very high. Studies done in pastoralist women are almost few. Therefore, the objective of this study was to assess the determinant factors of maternal death in the pastoralist area of Borena zone, Oromia region, Ethiopia.

**Methods:**

Community-based unmatched case-control study was conducted on 236 mothers (59 maternal deaths (cases) and 177 controls). The sample included pregnant women aged 15–49 years from September 2014 to March 2017. Data were collected using a structured questionnaire adapted from Maternal Death Surveillance and Response Technical Guideline, entered into the EpiData, exported into SPSS for analyses. Odds ratios (ORs) and 95% confidence interval (CI) were computed to determine contributing factors of maternal death and control potential confounding variables.

**Results:**

About 51 (86%) of all maternal deaths were due to direct obstetric causes. Of this, hemorrhage (45%), hypertensive disorders of pregnancy (23%), and obstructed labor (18%) were the leading direct causes of maternal deaths. Husbands who had no formal education were 5 times higher compared with their counterparts (AOR = 5.1, 95% CI: 1.6–16). Mothers who were not attending ANC were 5 times more at risk for death than those who attend (AOR 5.3, 95% CI 2.3–12.1). Mothers who gave birth at home/on transit were twice to die compared to health facility delivery (AOR 2.6, 95% CI 2.4–6) that were contributing factors of maternal deaths.

**Conclusions:**

Husband's level of education, lack of antenatal care, and home delivery were the factors contributing to maternal deaths in the zone. Frequent and tailored antenatal care, skilled delivery, and access to education also need due attention.

## 1. Background

It is the death of a woman during pregnancy or within 42 days of termination of pregnancy, irrespective of the duration and the site of the pregnancy. It can be from any cause related to or aggravated by the pregnancy or its poor management, but not from accidental or incidental causes [1].

According to the World Health Organization (WHO) document from Millennium Development Goals (MDG) to Sustainable Development Goals (SDG), an estimation of 303, 000 maternal deaths occurred worldwide from pregnancy and its complications which is equivalent to 830 mothers dying every day (more than 1 life lost every 2 minutes) in 2015. Nearly, all of these deaths are preventable in nature if appropriate interventions are taken [2]. Almost 99% of maternal deaths were reported from the developing regions that showed the largest discrepancy between developed and developing countries [3].

Among the developing countries, sub-Saharan Africa alone accounts for approximately 66% followed by Southern Asia, 22% of maternal deaths. Ethiopia is also categorized under the countries with high maternal mortality [3]. According to the Ethiopian Demographic and Health Survey (EDHS, 2016) report, the maternal mortality rate (MMR) in Ethiopia is 412/100,000 live births [4], which is considerably higher as of the World Health Organization and World Bank groups' classification [3].

Many studies also showed that the risk of maternal death is significantly associated with the occupation, age, education (of the mother and husband), substandard or no ANC service and visit, place of residency, maternal obstetrics characteristics (gravidity and parity), and preexisting problems [5–13].

On the contrary, most of the studies conducted on the causes of maternal death in Ethiopia are facility-based and among the agrarian community which might not actually reflect the contributing factors of maternal death occurring at the pastoralist community level [14, 15]. The pastoralist community is different from the rest of the population in access and utilization of health care services. Most of the pastoralist women give birth at home, unlike that of the rest of the women [16]. This is because they are mobile and living in remote areas of the country with dispersal in the settlement. The health system is not tailored to their way of living. Moreover, there is a shortage of literatures concerning to the determinant factors of maternal death in Ethiopia, particularly in the pastoralist area. Since there is no any study done at the pastoralist area of the Borena zone that determined the determinant factors of maternal death, the requirement of further study is unquestionable. Therefore, this study was aimed at assessing the causes and contributing factors of maternal death in the pastoralist area of the Borena zone.

## 2. Methods

The study was conducted in the pastoralist area of the Borena zone, one of the 18 zonal administrative divisions of the Oromia region, Ethiopia. The zonal capital, Yabelo town, is located 575 km from Addis Ababa in the south direction. The zone has 13 districts (10 pastoralists and 3 agrarian), 2 town administrations, and 248 rural and 15 urban kebeles (the smallest administrative unit).

Based on the 2007 GC Ethiopian Central Statistical Agency (CSA) report, the 2017 projected that total population of the zone is 1,365,753 with an estimation of 302,240 women of childbearing age (15–49 years), of which 47,391 of them (3.47% of total population) are expected to be pregnant [6].

Population density of the zone is assumed to be 23 people per km^2^, and 91% of them live in rural areas with arid and semiarid climate condition. Most of the rural kebeles and villages are very remote in terms of health access and facilities.

The zone has 3 hospitals (1 zonal and 2 district), 66 health centers, 217 health posts, and 92 private clinics. Only 35 (53%) of 66 health centers and 3 hospitals are providing basic emergency obstetrics care and two hospitals providing comprehensive emergency obstetrics care services.

A community-based unmatched case-control study was conducted among pregnant women who delivered between September 2014 and March 2017.

Cases were all women of the reproductive age group who died during pregnancy, delivery, and within 42 days after delivery between September 2014 and March 2017 while controls were all women in the reproductive age group who delivered including stillbirth and abortion, those alive within 42 days after delivery between September 2014 and March 2017.

### 2.1. Inclusion and Exclusion Criteria

Cases who fulfilled the standard case definitions of maternal death given by international classification of disease-10 (ICD-10) and controls who have a willingness to participate in the study were included. Of deaths, not related to pregnancy and/or beyond 42 days of termination of pregnancy were excluded from the study.

### 2.2. Sample Size Determination and Procedure

The sample size was determined by the two population proportion formula using Epi Info version 7 considering the following assumptions: 95% CI, 80% power, 1 case to 3 control ratios (1 : 3), percent of controls represented as 45.42%, and adjusted odds ratio of 2.594 (odds of rural to urban resident) from a case-control study done at the Jimma Referral Hospital, southwest Ethiopia [5]. So, a total of 216 (54 cases and 162 controls) sample size was determined. By adding 10% for the nonresponse rate (5 cases, 15 controls), 236 (59 cases and 177 controls) samples were included in the study.

Individual cases and controls fulfilling the inclusion criteria were selected retrospectively from the most recent death (for cases) and delivery including termination of pregnancy and still births (for control) until the determined sample size was achieved. All maternal deaths reported from September 2014 to March 2017 through Maternal Death Surveillance and Response (MDSR) was retrieved from verbal autopsy summary and facility-based abstraction form. Then, the sampling frame was prepared. For each selected case, three delivered mothers were interviewed as controls. But if more than three control mothers were eligible, simple random samplings were used.

Data were collected by four senior midwifery nurses and two health officers with the help of health extension workers (HEWs) as a local guide, using a structured questionnaire that adapted from the Federal Democratic Republic of Ethiopia, Ministry of Health MDSR Technical Guideline [7].

The questionnaire was translated into the local language, Afan Oromo. Maternal death information was collected from maternal death reporting format (VA summary form) and facility-based abstraction form. Controls' information was collected from women in the reproductive age group who gave birth or terminated the pregnancy in the study period. Data collectors were trained by the principal investigators for one day on the details of data collection instrument, interviewing techniques, and the importance of data quality and research ethics.

For study variables, see Table 1.

### 2.3. Data Processing and Quality Assurance

Questionnaires filled every day were reviewed and checked for completeness and consistence by the principal investigator for keeping quality. After data collection was completed, each filled questionnaire was coded by the principal investigators. The data were entered into EpiData version 3.1 and exported into SPSS version 20 computer software programs for cleaning and analyses. For each variable under the study, simple frequency was run and used to check for entry errors, missing values, and outliers. Any identified error was cross checked with the previously coded original questionnaires using the code number and then corrected accordingly.

### 2.4. Data Analysis

Following the data checking for any discrepancies, descriptive analysis was performed. Bivariate logistic regression analysis was done to decide whether there is an association between maternal death and different factors to select candidate variables for multivariate logistic regression.

Variables with a *p* value less than or equal to 0.25 or crude odds ratios show that significant association were entered into multivariate binary logistic regression to identify predictors of maternal deaths. *P* values of <0.05 and/or AORs with 95% CI interval not containing number 1 were taken as statistically significant. ORs, 95% CI, and *p* values were reported for all independent variables. Graphs and figures such as bar/pie charts and tables were used to present findings of the study. Delays and categories of delays were summarized from maternal death reporting format (VA summary) and facility-based abstraction form, which are developed based on the WHO delay modalities.

### 2.5. Operational Definitions

*Maternal death* is the death of a woman while pregnant or within 42 days of the termination of pregnancy, irrespective of the duration and the site of the pregnancy, from any cause related to or aggravated by the pregnancy or its management but not from accidental or incidental causes. *Direct obstetric death* is maternal deaths resulting from obstetric complications of pregnancy, labor, and puerperium period, whereas *indirect obstetric death* is a maternal death resulting from any previously existing disease aggravated by pregnancy or disease that developed during current pregnancy.

### 2.6. Ethical Consideration

Ethical clearance was obtained from the Institutional Review Board (IRB) at College of Medicine and Health Science of Hawassa University. Permission was also gained from the Borena zone health department and respective district heath offices of the study area. Informed verbal consent was obtained from individual study participants after briefing the risks and benefits of the study. Name of the study participants was not written on the questionnaire.

## 3. Results

### 3.1. Sociodemographic Characteristics of Study Participants

A total of 236 participants (59 cases and 177 controls) were included in this study.  Table 2 provides a detailed descriptive result of the study participants. The mean age of study participants was 28 for cases and 27 for controls. More than half (110 (57%)) of controls and nearly half (30 (51%)) of cases were in the age group of 25–34 years, and the least age group was 35–49 years old in both cases and controls.

Forty four (75%) of cases and 79 (45%) of controls mothers' husband were pastoralist. Ninety-six percent of study subjects were married. Most of the cases (93%) and more controls (64%) as well as their husbands had no formal education (Table 2).

### 3.2. Obstetric History of Study Participants

Out of all cases and controls as shown in Table 3, 17 (29%) of cases and 76 (43%) of controls were gravida 1-2 and 23 (39%) of cases were para 4 and above while 84 (47%) of controls were in parity 2-3.

Thirty four (58%) of cases did not have a history of antenatal care (ANC) follow-up, whereas majority (158 (89%)) of the controls had a history of ANC follow-up for index pregnancy. As to the delivery history, only 27 (46%) of the cases were delivered at health facility while majority (146 (82%)) of the control gave birth in the health facility. Similarly, for more than half, 32 (54%) of cases, their delivery was assisted by traditional birth attendants (TBAs) or their family/elders, while majority (147 (83%)) of controls were assisted by health workers (Table 3).

### 3.3. Obstetrics Risk and Complications

Table 4 shows that a total study participant, 15 (8.5%) of controls and 4 (7%) of cases, had a history of preexisting health problems before the last pregnancy. Most, 47 (79.6%) of the cases and 46 (26%) of the controls, had experienced problems during their antenatal/intranatal of the last child pregnancy. Vaginal bleeding and preeclampsia are the two leading causes of problems occurred during antenatal or intranatal of the last child pregnancy in the case populations.

Individuals who were not seeking care for their problems, not knowing the impact of illness in 11 (58%) of the cases and 4 (50%) of the controls, past good obstetric outcome at home in 3 (16%) of the cases and 2 (25%) of the controls, lack of transport in 3 (16%) of the cases, and no nearby health facility in 2 (10.5%) of the cases and 2 (25%) the controls were the main reasons why the care was not sought.

Conversely, 127 (72%) of the controls and 4 (7%) of the cases had not experienced problems during antenatal/intranatal of the last child pregnancy. Of 25 study participants who had preeclampsia, 15 (60%) of them were deceased. Vaginal bleeding occurred in 25 of the study participants, of which 20 (80%) of them were deceased.

### 3.4. Place of Death and State of Pregnancy at Time of Death

**Figure 1:**
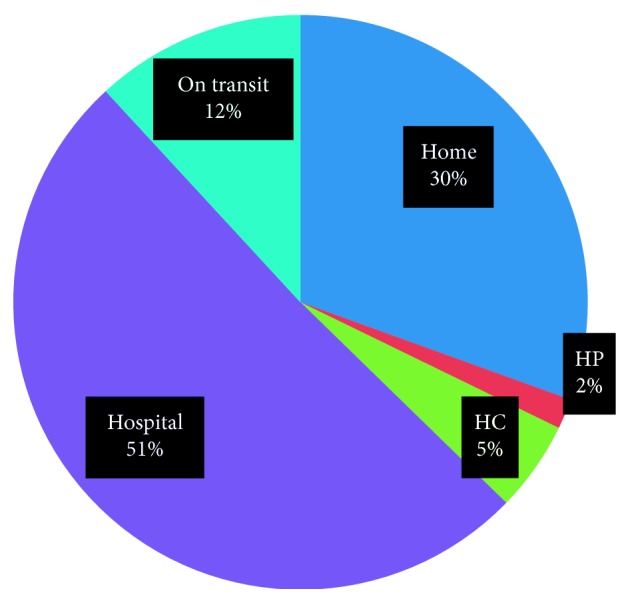
Maternal death by place among pastoralist pregnant women in the Borena zone of Oromia region, Ethiopia, 2017.

As Figure 1 shows, of the total 59 maternal deaths, nearly half (51%) of them was occurred in hospital while 18 (30%) occurred at home (Figure 1).

**Figure 2:**
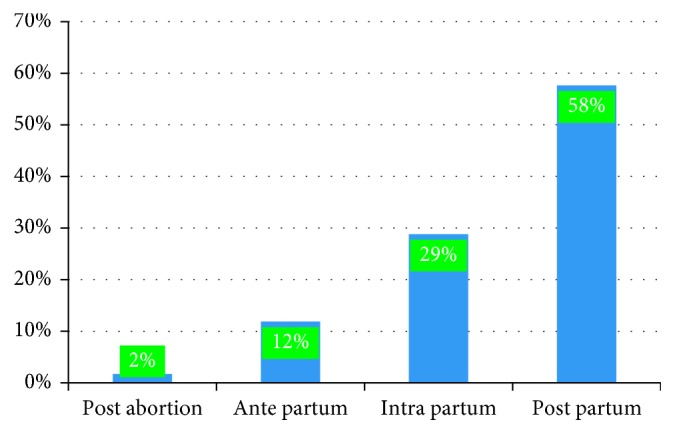
State of pregnancy at time of death, at pastoralist area of Borena zone, Oromia region, Ethiopia, 2017.

Figure 2 shows state of pregnancy at the time of death, 34 (58%) of them happened during the postpartum period and 17 (29%) occurred in intrapartum. Of the 34 deaths that occurred during the postpartum period, 18 (53%) of them occurred in hospital and 10 (29%) were at home.

Of 59 maternal deaths, most (41 (69.5 %)) of them were occurred during the gestational age of greater or equal to 37 weeks. Others (8 (14%)) were before 37 week of gestation. Among postpartum/posttermination of pregnancy-period deaths, 24 (70%) of them occurred within the first 3 days and 9 (26%) between 4 and 7 days. Almost all (97%) of the death occurring during postpartum/posttermination of the pregnancy period were within the first one week. Most (41 (69.5%)) of the deceased mothers were referred from another facility or their home (Figure 2).

### 3.5. Causes of Maternal Deaths

Out of 59 maternal deaths, 51 (86%) of them were due to direct obstetric causes while 8 (14%) were from indirect obstetric causes. From direct obstetric causes of deaths, hemorrhage was responsible for 23 (45%) of them followed by hypertension disorder of pregnancy (12 (23%)). The majority (17 (74%)) of reported hemorrhage deaths occurred in the postpartum period while intra- and antepartum hemorrhage accounted for 5 (22%) and 1 (4%), respectively.

Among 8 indirect obstetric causes of maternal deaths, 4 (50%) were due to anemia, 2 (25%) from malaria, and 12.5% from TB and hypertension each.

Table 5 provides descriptive statistics of other factors such as delay in recognizing and seeking care on time (delay 1), delays in reaching care (delay 2), and delay in receiving appropriate care timely (delay 3) during complication of a pregnancy at different levels (Table 5).

With regard to first delays, of the total 59 deceased mothers, 55 (93%) of them were delayed due to lack of a decision to go to health facilities to seek obstetric care and 88% of them also delayed because of failure of recognition of the problems. After deciding to go to health facilities, 46 (85%) of 54 were delayed referral from home.

With respect to second delays, out of 46, 29 (63%) of them faced transportation problems to reach the health facilities for receiving emergency obstetric cares. They went an average of 89 km to reach the nearest hospital. As to the means of transportations, among 59 deceased mothers 22 (37.3%) used the public transport, 29 (49.2%) by ambulance, 5 (8.5%) on foot, 3 (5%) by rented private cars.

Twenty five (57%) of 44 were delayed to arrive to the referred facilities. Lack of roads also complained among 17 (36%) of the cases.

Regarding the third delays, among 35 mothers referred to the next facility from another facility and 18 (51%) of them were delayed to arrive. From 34 mothers who arrived at health facilities, 21 (62%) were deceased due to absence of supplies (commonly, blood transfusion) and equipment at health facilities. Delayed management after admission was encountered in 10 (29%) of the deceased mothers.

### 3.6. Factors Contributing to Maternal Deaths

Sociodemographic and obstetric variables were analyzed using binary logistic regression methods separately. Age in years, place of residence, mothers and husbands' level of education, marital status were analyzed in bivariate analyses. Among the sociodemographic variables entered into the model, place of residence and husband education were remained significant. Secondly, obstetrical variables such as number of gravidity and parity, presence and place of ANC attendance, delivery place, type of birth attendant, any preexisting problems, and presence of antenatal/intranatal risk and types were also analyzed. Parity, ANC attendance, and delivery place have remained significant in bivariate analyses. Finally, variables significant during bivariate analysis were entered in the model for multivariate analysis and husband's education, ANC attendance, and delivery place that were the variables significantly associated with maternal death in a multivariate analysis model.

Table 6 shows that husbands who had no formal education were 5 times higher compared with their counterparts (AOR = 5.1, 95% CI: 1.6–16). Mothers who were not attending ANC were 5 times more at risk for death than those who attend (AOR 5.3, 95% CI: 2.3–12.1). Mothers who gave birth at home/on transit were more than twice to die compared to health facility delivery (AOR 2.6, 95% CI: 2.4–6).

## 4. Discussion

The results showed that more than 85% of maternal deaths in the zone were due to direct obstetric causes. This finding was higher than the result of systematic analysis study done by the WHO on the global causes of maternal death, which indicated that nearly 70% of maternal deaths were due to direct obstetric causes worldwide. Of which, hemorrhage and hypertensive disorder of pregnancy were the two leading causes of maternal deaths and more than 60% of the reported hemorrhage deaths were categorized as postpartum [8]. In our study, this is also true that nearly three-fourth of reported hemorrhage death occurred during the postpartum period. Other studies conducted at Gilgel Gibe field research center and among six rural districts of Tigray, northern Ethiopia, showed that nearly sixty percent were hemorrhage and hypertensive disorder of pregnancy were the leading causes in both studies [9, 10]. The results are lower than our study finding. This might be due to low accessibility and utilization of health service at the pastoralist area.

Deaths due to indirect obstetric causes accounted for 14% of all maternal deaths in the zone in the last three years. Among indirect obstetric causes, anemia contributed half, malaria one-fourth, and TB and preexisting hypertension 12.5% each of maternal deaths. This finding is less than the study conducted in six rural districts of Tigray, which reveals that nearly 38% of maternal deaths were from indirect obstetric causes such as anemia, malaria, and tuberculosis [10].

In this study, delays in reaching care (delay 2) for receiving emergency obstetric care were common. Sixty-three percent of deceased mothers were delayed to reach care for receiving emergency obstetric care at health facilities as a result of transportation problems. This is greater than the study done at Bahir Dar [11]. This may be because of the settlement of pastoral population that was highly dispersed compared to agrarian population. In addition, delayed arrival to referred facility in 57%, lack of facility within a reasonable distance in 43%, and lack of road in 36% were reported among deceased mothers in the study area. In terms of accessibility, on average, they travel 89 km distance to reach the nearest hospital is the evidence of inaccessibility.

Mothers whose husband had no formal education were 5 times more likely to die. This means that the probability of dying in mothers whose husband had no formal education been much higher than their corresponding ones. The finding of this study is consistent with a study done in the Lemo district of South Ethiopia [17] and Tanzania showed that mothers whose husbands did not have any formal education were twofold more likely to die compared to those whose husbands were from 1–7 school grades [18]. The reasons for this might be the husbands who had formal education would have a better understanding of information about the importance of maternal and child health services. Moreover, if the husband had formal education, he would more likely have awareness about obstetric danger signs, complication readiness, birth plan, on time referral during problem, and so on that reduces maternal deaths.

Mothers who did not attend ANC were 5 times more likely to die compared to those who attended ANC. This finding is in line with studies done in Kenyan tertiary hospital [12], and Mizan-Tepi University teaching and Bongs general hospital [13] and public hospitals in Mekelle town indicated that attending ANC can protect maternal death compared to those who did not attend ANC [14]. This might be because antenatal care is important in screening for any preexisting problems. In addition to that, if the pregnant mother fails to attend ANC, she might miss different services such as iron supplementation for prevention of anemia, TT vaccination for prevention of tetanus from her and new born, and different counseling such as birth preparedness and complication readiness, birth place, and postnatal service for both new born and herself. That is why attending ANC is considered as a protective factor or not attending ANC makes mothers at risk for death.

This study also found that mothers who gave birth at home more than two times are more likely to die compared to those who gave birth at health facility. This finding is also in line with the study done in Nigeria and Peru [19, 20]. This is because of mothers giving birth at home might not be assisted by skilled person and clean environment. Therefore, the risk of infection and bleeding and other related complications is high and cannot be managed urgently.

### 4.1. Limitations of the Study

Firstly, case population was taken from the previously collected verbal autopsy information and facility abstraction form; hence, bias may have been introduced during the classification of causes of deaths and contributed factor, and some important information might be missing. Secondly, since control populations were interviewed about the past situation, they might fail to remember some issues that may lead to recall bias.

## 5. Conclusion

In conclusion, majority of maternal deaths in the zone were due to direct obstetric causes. Hemorrhage and hypertension disorder of pregnancy were the two leading direct causes of obstetric deaths in the zone. Postpartum hemorrhage was leading causes of death from hemorrhage-related death. Anemia and malaria are the common indirect causes of maternal death. Husbands' education, attending ANC, and place of delivery were found as determinant factors of maternal death in the pastoralist area of the Borena zone. Therefore, frequent and tailored antenatal care service should be designed for rural and pastoralist community, and access to education also needs due attention. Educating pregnant women and their husbands about danger signs, including hemorrhage, is very crucial for the pastoralist community.

## Figures and Tables

**Table 1:** List of the study variables.

Dependent	Independent
Maternal death	(i) *Sociodemographic variable* Age, ethnicity, place of residence, occupation, religion, level of education, and monthly family income (ii) *Obstetric and delivery history* Parity, gravidity, antenatal care (ANC), place of delivery/death, delivery attendants, and number and place of ANC visited, (iii) *Preexisting problems* Hypertension, anemia, malaria, diabetes, HIV-positive tuberculosis infection, and others (iv) *Antenatal/intranatal problems* Preeclamsia/eclamsia, vaginal bleeding, multiple gestation, and malpresentation

**Table 2:** Sociodemographic characteristics of pregnant women who delivered between September 2014 and March 2017 at pastoralist area of Borena zone, Oromia region, Ethiopia (*n*=236).

Variables	Case, *n* (%)	Control, *n* (%)	Total, *n* (%)
*Age in years*			
15–24	17 (29)	58 (33)	75 (32)
25–34	30 (51)	101 (57)	131 (55)
35–49	12 (20)	18 (10)	30 (13)

*Place of residency*		
Rural	53 (90)	108 (61)	161 (68)
Urban	6 (10)	69 (39)	75 (32)

*Occupation of mother*			
Pastoralist	23 (39)	52 (29)	75 (32)
Farmer	5 (8)	17 (10)	22 (9)
Housewife	30 (51)	78 (44)	108 (46)
Merchant	1 (2)	12 (7)	13 (5)
Student	0 (0)	2 (1)	2 (0.8)
Unemployed	0 (0)	2 (1)	2 (0.8)
Government employee	0 (0)	13 (7)	13 (6)
Day laborer	0 (0)	1 (1)	1 (0.4)

*Occupation of husband*			
Pastoralist	44 (75)	79 (45)	123 (52)
Farmer	8 (14)	30 (17)	38 (16)
Merchant	3 (5)	23 (13)	26 (11)
Student	0 (0)	2 (1)	2 (1)
Unemployed	2 (3)	1 (1)	3 (1)
Government employee	1 (2)	27 (15)	28 (12)
Day laborer	1 (2)	15 (8)	16 (7)

*Educational status of mother*			
No formal education	55 (93)	114 (64)	169 (72)
Formal education	4 (7)	63 (36)	67 (28)

*Husband's education*			
No formal education	54 (92)	98 (55)	152 (64)
Formal education	5 (8)	79 (45)	84 (36)

*Marital status*			
Married	57 (97)	170 (96)	227 (96)
Widowed + divorced	2 (3)	7 (4)	9 (4)

**Table 3:** Obstetric history of pregnant women who delivered between September 2014 and March 2017 at pastoralist area of Borena zone, Oromia region, Ethiopia.

Variables	Cases, *n* (%)	Controls, *n* (%)	Total, *n* (%)
*Gravidity (n* *=* *236)*			
1-2	17 (29)	76 (43)	93 (39)
3-4	23 (82)	59 (33)	82 (35)
5+	19 (32)	42 (24)	61 (26)

*Parity (n* *=* *236)*			
0-1	16 (27)	28 (16)	44 (19)
2-3	20 (34)	84 (47)	104 (44)
4+	23 (39)	65 (37)	88 (37)

*ANC attended (n* *=* *236)*			
Yes	25 (42)	158 (89)	183 (78)
No	34 (58)	19 (11)	53 (22)

*Number of ANC visits (n* *=* *183)*			
1	7 (28)	9 (6)	16 (9)
2	14 (56)	24 (15)	38 (21)
3	3 (12)	51 (32)	54 (30)
4 and above	1 (4)	74 (47)	75 (41)

*Place of ANC visits (n* *=* *183)*			
Health Post	9 (36)	22 (14)	31 (17)
Health center + hospital	16 (64)	136 (86)	152 (83)

*Place of delivery (n* *=* *236)*			
Home	29 (49)	28 (16)	57 (24.1)
In transit to health facility	3 (5)	3 (2)	6 (2.5)
Health facility	27 (46)	146 (82)	173 (73.4)

*Birth assisted by (n* *=* *236)*			
Family/elder + TBA	32 (54)	27 (15)	59 (25)
HEWs	2 (3)	3 (2)	5 (2)
Health workers	25 (42)	147 (83)	172 (73)

**Table 4:** Preexisting problems and/or antenatal/intranatal risk during the last child pregnancy of pregnant women who delivered between September 2014 and March 2017 at pastoralist area of Borena zone, Oromia region, Ethiopia.

Variables	Cases, *n* (%)	Controls, *n* (%)	Total, *n* (%)
*Preexisting health problems before last pregnancy*			
Yes	4 (7)	15 (8.5)	19 (8)
No	55 (93)	162 (91.5)	217 (92)

*Types of preexisting problems*			
Hypertension	2 (50)	10 (67)	12 (63)
Malaria	1 (25)	4 (27)	5 (26)
Tuberculosis	1 (25)	0	1 (5)
PID	0	1 (6)	1 (5)

*Antenatal/intranatal risk during the last pregnancy*			
Yes	47 (79.7)	46 (26)	93 (39.4)
No	12 (20.3)	131 (74)	143 (59.6)

*Types of antenatal/intranatal risk* ^1^			
Preeclampsia	15 (32)	10 (22)	25 (27)
Vaginal bleeding	20 (43)	5 (11)	25 (27)
Previous cesarean section delivery	0	4 (9)	4 (4)
Multiple gestation	2 (4)	1 (2)	3 (3)
Abnormal lie/presentation	7 (15)	4 (9)	11 (12)
Anemia	8 (17)	17 (37)	25 (27)
Malaria	2 (4)	5 (11)	7 (8)
Unplanned pregnancy	0	1	1 (1)
Others^2^	5 (11)	5 (11)	10 (11)

^1^Multiple responses possible. ^2^CPD, infection; IUFD, obstructed labor; PROM, retained placenta and uterine rupture.

**Table 5:** Three delays for maternal death and its elements at pastoralist area of Borena zone, Oromia region, Ethiopia, 2017.

Delays and its elements^*∗*^	Frequency (%)	
	Yes	No
*Delay 1 (delay in recognizing and seeking care)*		
Traditional practices (*n* = 59)	39 (66)	20 (34)
Lack of decision to go to a health facility (*n* = 59)	55 (93)	4 (7)
Failure of recognition of the problem (*n* = 59)	52 (88)	7 (12)
Delayed referral from home (*n* = 54)	46 (85)	8 (15)

*Delay 2 (delays in reaching care)*		
Delayed arrival to referred facility (*n* = 44)	25 (57)	19 (43)
Lack of roads (*n* = 47)	17 (36)	30 (64)
Lack of transportation (*n* = 46)	29 (63)	17 (37)
No facility within reasonable distance (*n* = 46)	20 (43)	26 (56)
Lack of money for transport (*n* = 46)	8 (17)	38 (83)

*Delay 3 (delay in receiving appropriate care)*		
Delayed arrival to the next facility from another facility on referral (*n* = 35)	18 (51)	17 (49)
Delayed or lacking supplies and equipment (*n* = 34)	21 (62)	13 (38)
Delayed management after admission (*n* = 34)	10 (29)	24 (71)
Human error or mismanagement (*n* = 33)	1 (3)	32 (97)

^*∗*^Multiple responses possible.

**Table 6:** Bivariate and multivariate logistic regression analysis of factors associated with maternal death at pastoralist area of Borena zone, Oromia region, Ethiopia, 2017.

Variables	Case (%), *n* = 59	Control (%), *n* = 177	COR (95% CI)	AOR (95% CI)	*P* value
*Place of residence*					
Urban	6 (10)	69 (39)	1	1	
Rural	53 (90)	108 (61)	5.6 (2.3–13.8)	2.17 (0.7–6.8)	0.18

*Husband's education*					
No formal education	54 (92)	98 (55)	8.70 (3.3–22.8)	5.13 (1.6–16)^*∗*^	≤0.001
Formal education	5 (8)	79 (45)	1	1	

*Parity*					
0-1	16 (27)	28 (16)	1.6 (0.7–9.1)	2.74 (0.9–8.6)	0.8
2-3	20 (34)	84 (47)	0.7 (0.34–4.3)	1.64 (0.67–3.9)	0.28
4+	23(39)	65(37)	1	1	

*ANC attendance*					
Yes	25 (42)	158 (89)	5.48 (2.4–12)^*∗*^	5.3 (2.3–12.1)^*∗*^	0.004
No	34 (58)	19 (11)	1		

*Place of delivery*					
Home/on transit	32 (54)	31 (17)	5.78 (2.2–11.4)^*∗*^	2.62 (2.4–6)^*∗*^	≤0.001
Health facility	27 (46)	146 (83)	1	1	

^*∗*^OR (95% CI) shows significant associations at *P* < 0.05 and 1 represents the reference category.

## Data Availability

The datasets generated and/or analyzed during the current study are available from the corresponding author on reasonable request.
